# Role of Napsin A and Survivin Immunohistochemical Expression in Bronchogenic Adenocarcinoma

**DOI:** 10.31557/APJCP.2020.21.11.3345

**Published:** 2020-11

**Authors:** Maha Elsayed Mohammed Salama

**Affiliations:** *Department of Pathology, Faculty of Medicine, Cairo University, Cairo, Egypt. *

**Keywords:** Napsin A, survivin, bronchogenic, adenocarcinoma

## Abstract

**Background:**

Lung cancer, being the leading cause of cancer deaths with most patients diagnosed at a late stage, represents a major burden in developing countries especially with both air pollution and tobacco use increasing. With the evolution of new, successful therapies that target lung adenocarcinoma, it became of utmost importance to diagnose lung adenocarcinoma. Despite considering TTF-1 as the predominant marker for identifying lung adenocarcinoma but it has limited sensitivity and specificity, which means that its expression decreases in relation to the degree of tumor differentiation.

**Aim of work:**

this study intended to evaluate the use of Napsin A in lung adenocarcinoma, and observe if it can withstand along the different lines of tumor differentiation and Survivin as a marker of poor prognosis.

**Materials and Methods:**

Forty paraffin blocks of bronchogenic carcinoma were collected and studied immunohistochemically against Napsin A and Survivin.

**Results:**

There was a statistically significant relation between Napsin A reactivity and tumor grade as 72% of grade II as well as all cases of grade I were strongly positive compared to none of grade III cases. Another statistically significant relation between Survivin reactivity and tumor grade was observed as all grades I and II cases showed labeling index <10%, while all grade III cases showed labeling index >10%.

**Conclusion:**

Napsin A is a good prognostic marker while Survivin stands as a poor one for lung adenocarcinoma with a statistically inverse relation between the two, which means that Napsin A can’t be used as a marker for diagnosing poorly differentiated tumors.

## Introduction

Lung cancer is still the commonest cancer and the leading cause of cancer deaths, unfortunately, most patients are diagnosed at an advanced stage. Tobacco use remains the most important risk factor, with other risk factors emerging, such as air pollution. With both air pollution and tobacco use increasing in most developing countries, there is a worrying potential for an increase in lung cancer burden in these places especially that they can’t afford screening tools or expensive treatment methods (Finke et al., 2020). Lung carcinoma has been divided into small and non-small cell carcinoma. But, with the development of new, successful treatments it is essential to differentiate non-small cell carcinoma into histologic types, especially because of new, successful therapies that target lung adenocarcinoma. Although TTF-1 is the predominant marker for lung adenocarcinoma but it has limited sensitivity and specificity (Bradely et al., 2012). Moreover, its expression decreases with the decrease in the tumor differentiation. Thus, emerges the urge to search for a surrogate marker with greater sensitivity and specificity for lung adenocarcinoma, along the lines of tumor differentiation. Napsin A is a functional aspartic proteinase that may be an alternative marker for primary lung adenocarcinoma (Stoll et al., 2010). It is one of the pepsin family involved in the maturation of surfactant protein B and is found primarily in lung and kidney. Lack of its expression in tumor cells is a poor prognostic marker in pulmonary adenocarcinoma (Jianghua et al., 2020). Although Napsin A proved to be more specific and sensitive than TTF1 in lung adenocarcinoma but can it detect poorly differentiated tumors? This has been our research question which was achieved by studying the reactivity of Napsin A and survivin in lung adenocarcinoma cases. Survivin is an inhibitor of apoptosis which is widely expressed in poorly differentiated tumors and is markedly related to poor prognosis (Zhou et al., 2020).

## Materials and Methods

The current study was conducted on paraffin blocks of 40 cases diagnosed with primary bronchogenic adenocarcinoma carcinoma, confirmed by TTF1 positivity, either obtained from the data sheets of the patients or done at our costs. 

Serial sections were cut at 4 micron thickness from each paraffin block for histopathological evaluation, as well as immunohistochemical staining.

*Immunohistochemical staining*

• For the assessment of Napsin A expression, a representative slide from each case was stained using an antibody (rabbit polyclonal antibody, clone 352A-78, ready to use, CELL MARQUE, DARMSTADT, GERMANY). As positive control, a section of normal lung was employed. 

• For the assessment of Survivin expression, a representative slide from each case was stained using an antibody (mouse monoclonal IgG2a (D-8): sc-17779 provided at 200 µg/ml, SANTA CRUZ BIOTECHNOLOGY, OR., USA). As positive control, a section of intestine was employed.

*Immunohistochemical evaluation*

• Napsin A: Staining intensity was evaluated as follows: negative: no staining, weak positive: minimal, patchy cytoplasmic staining and strong positive: moderate to intense-brown, cytoplasmic staining (Bradely et al., 2012).

• Survivin: To determine positive staining of cytoplasmic survivin, tumors were classified into two groups based on the percentage of positively stained cells, labeling index (LI): >10%, positive staining; and <10%, negative staining (Hirano et al., 2015).

*Statistical Analysis*

Data were statistically described in terms of means and standard deviation or medians and ranges as well as percentages. Comparison between the groups was done by Chi square and Fisher exact tests. All statistical calculations were done using computer program SPSS (Statistical Package for the Social Science; SPSS Inc., Chicago, IL, USA) version 25. p values less than 0.05 was considered statistically significant.

## Results

The present study was done on 40 cases of bronchogenic adenocarcinoma. The age of the studied cases ranged from 30 up to 80 years with mean age 57.8. Our study showed male predominance (70%). Seventy percent of our cases were smokers. Concerning the grade, most of our cases were of grade II (62.5%), followed by grade III (25%) then grade I (12.5%). All differentiated adenocarcinoma cases included in the current study were of acinar subtype. 

*Napsin A expression*

**Figure 1:**
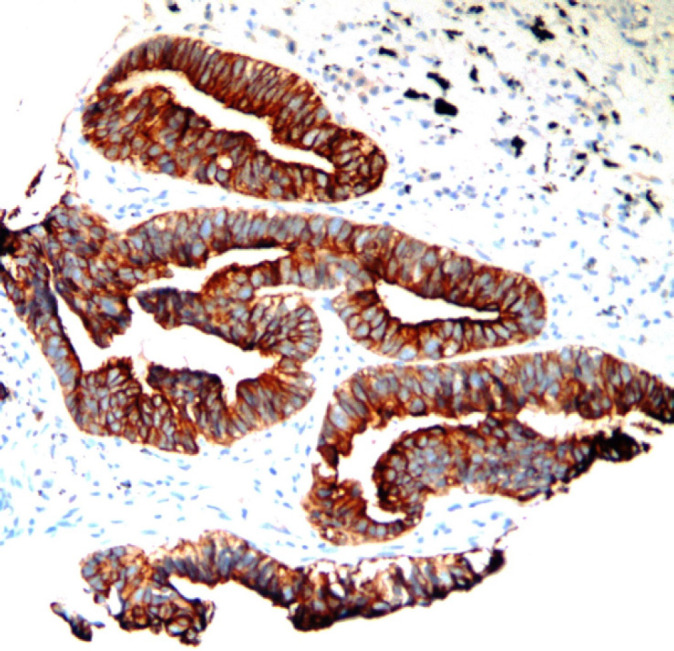
A Case of Bronchogenic Adenocarcinoma Grade I Showing Strong Napsin A Immunoexpression (Napsin A x200 Original Power).

**Figure 2:**
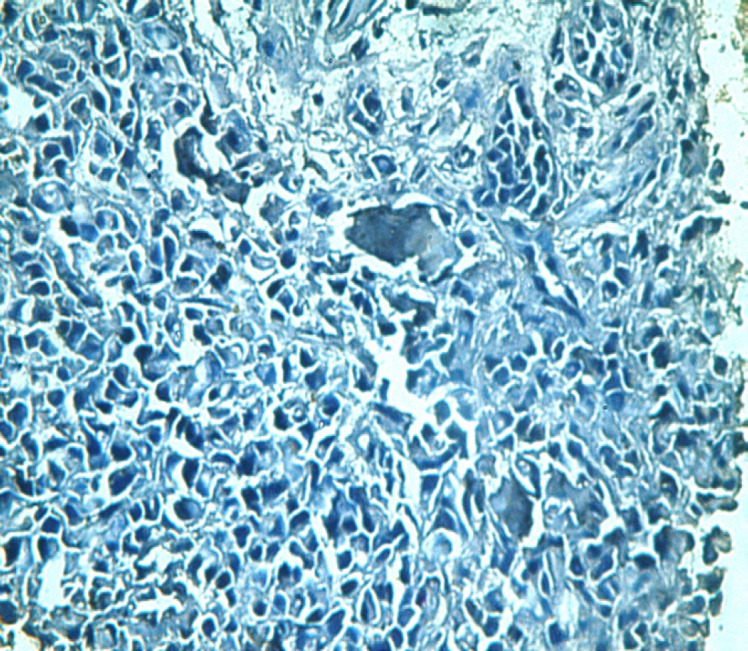
A Case of Bronchogenic Adenocarcinoma Grade III Showing Negative Napsin A Immunoexpression (Napsin A x400 Original Power)

Napsin A was positively stained in 30 cases. All cases diagnosed as grades one and two showed Napsin A positive staining. Regarding staining intensity, 72% of grade II cases were strongly positive (18/25) while the remaining 28% of cases were weakly stained (7/25). As for grade I, all the 5 cases showed strong staining (Figure 1). None of the studied grade III cases showed any Napsin A staining (Figure 2). These results were statistically significant.

No statistically significant relation could be found between Napsin A expression and any of the other patients’ clinicopathological parameters like age, sex and smoking history.

*Survivin expression*

**Figure 3:**
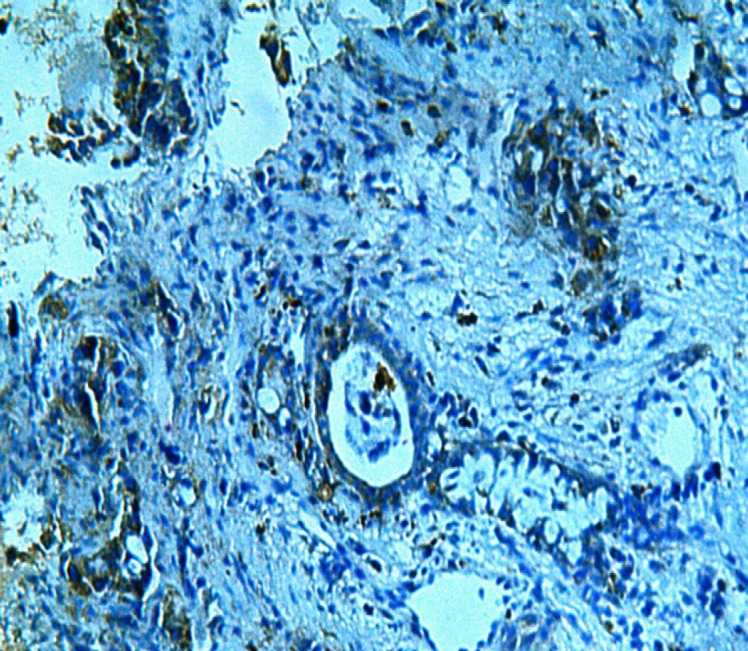
A Case of Bronchogenic Adenocarcinoma Grade II Showing Survivin Immunoexpression Labeling Index <10% (Survivin x200 Original Power)

**Figure 4:**
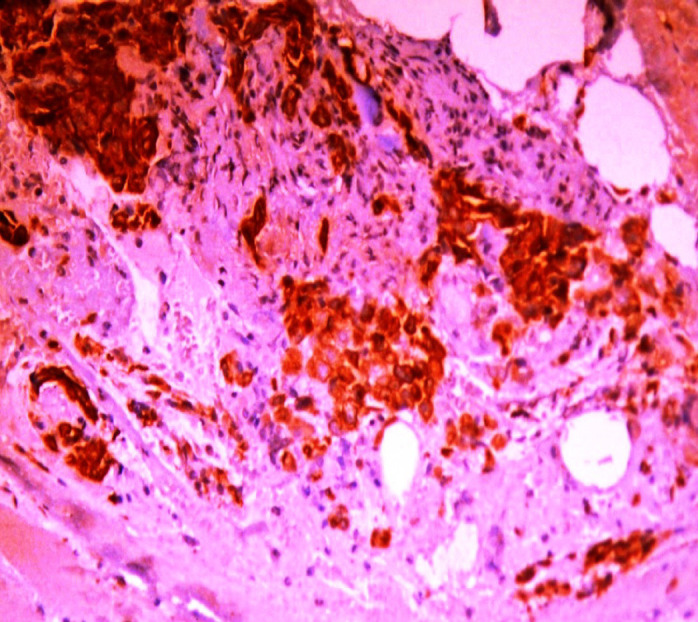
A Case of Bronchogenic Adenocarcinoma Grade III Showing Survivin Immunoexpression Labeling Index >10% (Survivin x200 Original Power)

Survivin was positively stained in all the 40 studied cases. Thirty cases showed labeling index less than 10% which include 100% of grades I and II (Figure 3) cases. The remaining 10 cases showed labeling index more than 10% which represent all grade III cases (Figure 4). These results were statistically significant.

No statistically significant relation could be found between survivin expression and any of the other patients’ clinicopathological parameters like age, sex and smoking history.

When comparing Napsin A expression intensity with survivin expression we found that there was a statistically significant inverse relation between the two as shown in Table 1. 

**Table 1:** Relation between Napsin A Staining Intensity and Survivin Labeling Index (LI)

Number of cases	Napsin A	Survivin		P value
		<10% LI	>10% LI	
10	Negative	0	100%	<0.05
7	Weak	100%	0	
23	Strong	100%	0	
Total 40 cases				

## Discussion

Lung cancer, being the leading cause of cancer deaths with most patients diagnosed at a late stage, represents a major burden in most developing countries especially with both air pollution and tobacco use increasing (Finke et al., 2020). With the evolution of new, successful therapies that target lung adenocarcinoma in particular, it became of utmost importance to be able to diagnose lung adenocarcinoma. Despite considering TTF-1 as the predominant marker for identifying lung adenocarcinoma but it has limited sensitivity and specificity, which means that its expression decreases in relation to the degree of tumor differentiation (Bradely et al., 2012). Therefore in this research we tried to evaluate the use of another marker with greater sensitivity and specificity for lung adenocarcinoma, and observe if it can withstand along the different lines of tumor differentiation. Napsin A was our target for study, especially that it is more sensitive and specific than TTF1 (Jianghua et al., 2020). Survivin was also included in our study as a marker of poor prognosis that basically increases its expression in lower differentiated tumors. 

Through studying 40 cases of lung adenocarcinoma, Napsin A was positively stained in 30 cases which represent all cases diagnosed as grades one and two. Regarding staining intensity, 72% of grade II cases were strongly positive (18/25) and the entire 5 grade I cases showed strong staining. None of the studied grade III cases showed any Napsin A staining. These results showed a statistically significant relation between Napsin A and tumor differentiation that is Napsin A is overly expressed in higher differentiated tumors. Our results go with those stated by Lee et al., (2012) who based on their results concluded that absence of napsin A was an independent prognostic factor for reduced survival time. Same conclusions were stated by Ma et al., (2015) who also recommended that it should be routinely performed in postoperative lung adenocarcinoma patients to determine the prognosis. 

In this study, Survivin was positively stained in all the 40 cases included. There was a statistically significant relation found between Survivin LI and tumor differentiation as 100% of grades I and II cases showed LI less than 10 compared to 100% of grade III cases showed labeling index more than 10%. Going with our study were the results concluded by Meng et al, (2012) and Duan et al., (2016) who stated that Survivin could serve as an important biomarker for lung carcinoma progression. In contrast to our results Atikcan et al., (2006) found no significant relation between Survivin and tumor grade so they couldn’t consider Survivin as a poor prognostic marker. 

When comparing Napsin A expression intensity with survivin LI, which to our knowledge has not been discussed before, we observed a statistically significant inverse relation between the two which was expected but not hoped for. We wished that Napsin A could stand as a marker for lung adenocarcinoma even in poorly differentiated tumors which was not achieved in our study. This could be attributed to our small sample size or even the small number of grade III cases included in this study (10/40). Other researchers are encouraged to continue in this field. 

From this study we conclude that Napsin A is a good prognostic marker while Survivin stands as a poor one for lung adenocarcinoma with a statistically significant inverse relation between the two. This means that according to our study Napsin A cannot be used as a marker for diagnosing poorly differentiated bronchogenic adenocarcinomas.
